# Mycotoxins in Feed and Food and the Role of Ozone in Their Detoxification and Degradation: An Update

**DOI:** 10.3390/toxins12080486

**Published:** 2020-07-30

**Authors:** Giuseppe Conte, Marco Fontanelli, Francesca Galli, Lorenzo Cotrozzi, Lorenzo Pagni, Elisa Pellegrini

**Affiliations:** Department of Agriculture, Food and Environment, University of Pisa, Via del Borghetto 80, 56124 Pisa, Italy; giuseppe.conte@unipi.it (G.C.); marco.fontanelli@unipi.it (M.F.); francesca.galli@unipi.it (F.G.); lore.pa@outlook.it (L.P.); elisa.pellegrini@unipi.it (E.P.)

**Keywords:** crop systems, pre- and post-harvest strategies, decontamination, ozonation

## Abstract

Mycotoxins are secondary metabolites produced by some filamentous fungi, which can cause toxicity in animal species, including humans. Because of their high toxicological impacts, mycotoxins have received significant consideration, leading to the definition of strict legislative thresholds and limits in many areas of the world. Mycotoxins can reduce farm profits not only through reduced crop quality and product refusal, but also through a reduction in animal productivity and health. This paper briefly addresses the impacts of mycotoxin contamination of feed and food on animal and human health, and describes the main pre- and post-harvest systems to control their levels, including genetic, agronomic, biological, chemical, and physical methods. It so highlights (i) the lack of effective and straightforward solutions to control mycotoxin contamination in the field, at pre-harvest, as well as later post-harvest; and (ii) the increasing demand for novel methods to control mycotoxin infections, intoxications, and diseases, without leaving toxic chemical residues in the food and feed chain. Thus, the broad objective of the present study was to review the literature on the use of ozone for mycotoxin decontamination, proposing this gaseous air pollutant as a powerful tool to detoxify mycotoxins from feed and food.

## 1. Overview of the Toxic Effects of Mycotoxins in Animals and Humans

Climate change is expected to negatively affect all dimensions of food and nutrition security and safety—including availability, access, utilization, and quality of principal food commodities—which are key aspects of food systems that are also challenged by the predicted increase in the global population [1,2]. Agriculture will be affected both directly, due to changes in global weather patterns (e.g., high temperature and modified rainfall amount and distribution), and indirectly, through diseases and pests, eventually leading to decreased yields and increased mycotoxin contamination [3,4].

Mycotoxins are chemical compounds synthesized as secondary metabolites by some filamentous fungi. The mycotoxins of most agricultural relevance are aflatoxins from *Aspergillus* spp., ochratoxin A (OTA) from *Aspergillus* spp. and *Penicillium* spp., fumonisins, type A trichothecenes (HT-2 toxin (HT-2) and T-2 toxin (T-2)) and type B trichothecenes from *Fusarium* spp., and patulin from *P. expansum* [5]. They vary in structure and can cause toxicity in a number of animal species. Metabolic and cellular disorders leading to various health impairments (e.g., reduced feed intake, nutrient absorption, and body weight; immunosuppression; reproductive syndromes; enlarged livers and kidney damages; subcutaneous and enteric hemorrhage and myocardial lesions; depression; and even death) were observed in poultry due to aflatoxins and OTA [6,7,8], in swine (the most sensitive species to mycotoxins) due to aflatoxin B1 (AFB_1_) [9], in sows due to zearalenone (ZEA) and T-2 toxin [10,11]; in horses due to AFB_1_ and moniliformin toxins [12]; and in ruminants (although they are considered less susceptible to mycotoxins than other animals, especially sheep and goats) due to aflatoxin mixtures [13,14,15].

Some mycotoxins have an elevated carry over rate from feed to milk, likely contributing to mycotoxin intake in human populations, which are also threatened [3]. The health threats of mycotoxins to humans have been reviewed largely in recent years (e.g., [16,17]). A broad variety of foods in the human diet can be contaminated by mycotoxins at different stages of the food chain, both pre- and post-harvest [18,19]. The major classes of mycotoxins affecting humans include AFB_1_ and aflatoxin B2 (AFB_2_), the strongest hepatocarcinogenic molecule known, also showing genotoxic properties, as evaluated by the World Health Organization (WHO)-International Agency for Research on Cancer in 1993. Moreover, the hydroxylation of the AFB_1_ and AFB_2_ involves the production of aflatoxin M1 and M2. Other major human mycotoxicoses have resulted from exposure to ergot, tricothecenes, ochratoxins, 3-ninotropropionic acid, ZEA, and fumonisins [17]. In addition, masked mycotoxins (produced by fungi but modified by plant enzymes during the infection stages) pose a major concern in food and feed as they are not identified and detected by the usually employed detection techniques [20]. Although toxicity data are scarce, the conversion of a masked mycotoxin to its free form may lead to increased bioavailability of mycotoxin and may pose a potential risk to human and animal health. Generally, clinical symptoms are vomit, diarrhea, hemorrhage, breathing difficulty, chest pain, blisters, headache, and fatigue, which can even lead to death [21]. Mycotoxicoses may be classified as acute or chronic: acute toxicity occurs quickly with an evident toxic response, while chronic toxicity shows a low-dose exposure over a long time period, resulting in irreversible effects [17,22]. The greatest risk of mycotoxins for animals and humans is commonly the consequence of chronic dietary exposure.

Because of their high toxicological impacts on both animal and human health, mycotoxins have received significant consideration by the Food and Agriculture Organization of the United Nations (FAO) and WHO, leading to the definition of strict legislative thresholds and limits in many areas of the world. Differently, in several African countries, the consumption of mycotoxin-contaminated foods is still a significant risk, especially for children, immunocompromised people, and rural populations [3]. However, numerous developing countries have realized that controlling and reducing the contamination of mycotoxins in food will decrease pressure on health-care systems, and enhance competitive advantage in exports. Regulatory agencies have established limits to keep under control the levels of mycotoxins in animal feed. Limits range from below one to thousands µg kg^−1^, depending on the mycotoxin, the food/feed commodity, and the country taken into account (e.g., [23,24]). The legislation applicable in the EU to products devoted to livestock feed is very strict and can block exports of feed from developing countries to their European trading partners [25]. Nonetheless, the legislation on mycotoxins does not consider the often reported and worrying scenario of multi-mycotoxin contamination of single commodities and animal feed [26]. In addition, regulations and recommendations for masked mycotoxins are completely missing, although since 2010, the FAO and the WHO have started to carry out risk assessments [27].

The relative resilience of various food and feed chains has become a major topic in the context of mycotoxin contamination levels. From a socio-economic perspective, losses due to mycotoxins are diverse and can be associated to multiple factors such as reduced yields, decreased nutritional value of feed and food, or increasing management costs due to mycotoxin issues. Several years ago, some authors in the United States have tried to calculate the mean economic annual costs at the farm gate of cereal crop losses due to aflatoxins and fumonisins [18]. In Europe, there is very limited data on the economic costs of mycotoxins, and this shows how it is difficult to make reliable and detailed estimates of the indirect costs linked to mycotoxin consequences [22]. Farmers directly experience a quantity and quality reduction of their products (be it for animal feed or human consumption) [28,29]. Moreover, animal breeders are affected because mycotoxins (as shown in detail in the following sections) can reduce animal production due to feed refusal or animal diseases. In addition, mycotoxins can reduce farm profits through a reduction in animal productivity and health. On top of the production loss, the increasing medical cost for mycotoxicosis treatments adds further economic damages. Other side costs are related to finding alternative feeds by the feed industry, to veterinary costs, to the design of an adequate management of contaminated supplies through protocols and remediation solutions. Adding to the complexity of the issue, feeds, forages, and food products can become contaminated with mycotoxins in the field, during harvest, during drying and transport as well as during storage.

Although the mere isolation of toxic fungal species is not sufficient to define the presence of mycotoxins, an interaction between different physical, chemical, and biological factors affecting fungal colonization and production of the mycotoxins has been reported [30]. Physical factors include environmental conditions such as temperature, relative humidity (RH), insect infestation and other associated factors, which at specific rates are known to favor fungal colonization and mycotoxin production [31]. However, optimal environmental conditions for fungal development are not necessarily optimal for toxin production [32]. Furthermore, such environmental constraints may stress plant species, making them even more susceptible hosts for toxic fungi. Biological factors are mainly related to the interactions between the colonizing toxic fungal species and the host, thus including features such as fungal species, strain specificity, strain variation, and instability of fungal toxic properties. Moreover, insect damage influences the extent of mycotoxin contamination [33,34]. The regulation of the environmental conditions mentioned above is an option to cope with mycotoxin colonization and production. Such regulation may occur by adopting different pre- and post-harvest control systems including agronomic, chemical, and physical methods. 

Overall, the control of mycotoxin contamination follows two strategies: prevention of their production (e.g., fungal or microbial inactivation) and detoxification (e.g., mycotoxin degradation) [4]. The present review paper firstly summarizes the various pre- and post-harvest systems to control the levels of mycotoxins, including genetic, agronomic, biological, chemical, and physical methods; and then gives support to ozone (O_3_) use not only as a food-sanitizing agent in coping with food decay during storage, but primarily for its potential in the inactivation of toxic fungi and in the detoxification of mycotoxins from feed and food.

## 2. Systems for the Control of Mycotoxins in Feed and Food

**Figure 1:**
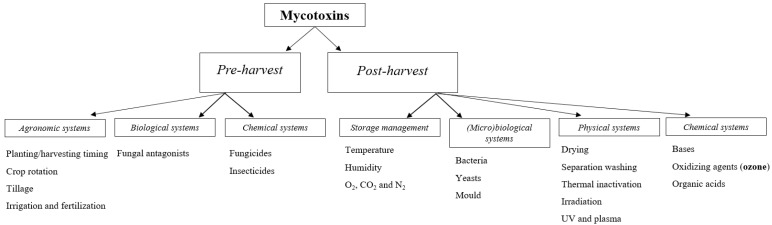
Pre- and post-harvest systems for the prevention and decontamination of mycotoxins in the food and feed chain.

The strategies developed to limit the accumulation of mycotoxins in feed and food are numerous and can be classified according to the intervention moment (i.e., pre- or post-harvest; [4,35]). These strategies are based on various systems such as agronomic, biological, chemical, and physical systems, as described in depth in the following paragraphs (Figure 1).

### 2.1. Pre-Harvest Strategies

The pre-harvest control of mycotoxins is based on the control of contamination levels in crops to be used as food and feed components. In general, these systems are based on preventive strategies, which aim to avoid the development of contamination, operating on the predisposing factors that facilitate the production of mycotoxins.

#### 2.1.1. Agronomic Systems

Agronomic practices such as planting/harvesting timing, crop rotation, tillage, and management of irrigation, highly affect the mycotoxin contamination of crops in the field [34]. Early harvesting decreases fungal infection of crops and consequent contamination of harvested products. Rachaputi et al. [35] found lower aflatoxin concentrations and higher gross returns (up to 27%) resulting from early harvesting and threshing of groundnuts. Farming systems can also affect the community structure composition of aflatoxin-producing fungi, as reported by Sserumaga et al. [36] in pre-harvest maize across Uganda (23 major maize-growing districts in eight agro-ecological areas). Crop rotation and intercropping can reduce the mycotoxin contamination in maize and groundnut, and in conservation agriculture (e.g., permanent soil cover and minimum soil disturbance) can decrease the risk of contamination [37]. Monoculture of maize under conventional tillage compared to no-tillage monoculture and no-tillage two- or three-year rotation (consisting of maize/cowpea and maize/cowpea/babala, respectively) showed no significant differences in terms of fumonisin contamination in South Africa [38]. Baliukonienè et al. [39] found a higher contamination of aflatoxins and deoxynivalenol (DON) in cereals from no-tillage farming systems than conventional farming systems (+14 and +24%, respectively). However, the toxin content of grain was not considerably influenced by the different tillage systems. These studies partially contradicted the review of Champeil et al. [40], who found that deep tillage, the choice of the preceding crop in the rotation and the choice of appropriate cultivar were the best cultural practices to prevent DON, nivalenol and ZEA. Dill-Macky et al. [41] found moldboard plowing the best soil management strategy to control Fusarium head blight (FHB) in wheat. 

Different management strategies for crop cultivation have also been compared for their effects on mycotoxin contamination. Góral et al. [42] compared organic and conventional winter wheat cultivars focusing on FHB and type A trichothecenes (HT-2, T-2, T-2 tetraol, T-2 triol, diacetoxyscirpenol and scirpentriol). FHB severity was at a similar level in both systems. *Fusarium* colonization of kernels was higher for the organic system than the conventional one. Studies conducted in Northern and Central Italy (in 2010 and 2011) on maize and wheat have not shown differences between organic and conventional farming systems in terms of the presence of mycotoxin-producing fungi. The incidence of *Fusarium* spp. was similar in conventional and organic wheat, but was significantly different in maize. *Aspergillus* and *Penicillium* incidence was linked to weather more than farming system; 2010 was the most favorable for *Fusarium* species (with 10 times the incidence of 2011; [43]). Cereals from organic farming systems in Norway resulted to be less infested by *F. graminearum* and *F. langsethiae* if compared to cereals from conventional farming systems [44]. These results are probably due to the less intense rotations and the lower input of organic farming systems. Similar results were obtained by other authors [45,46]. Similar contamination levels of ochratoxic fungi in conventional and organic coffee beans were found by Rezende et al. [47] in Brazil. Recently, Labanca et al. [48] evaluated the effect of different farming systems (i.e., conventional and organic cultivation) on mycotoxin levels in *Zea mays*, and similar contamination levels of AFB_1_ were found.

The use of modern technologies within a precision farming system could effectively help to prevent chemical contaminants like mycotoxins. Nazarizadeh et al. [49] described a precision livestock farming system for a real time monitoring of animals’ health status in order to enhance the safety of the meat chain. The use of a low-cost multispectral sorter was used to select and remove the maize kernels which were contaminated by aflatoxins and fumonisins in Kenya (the machine was calibrated by building a mathematical model relating reflectance at nine distinct wavelengths (470–1550 nm) to mycotoxin levels of single kernels [50]).

#### 2.1.2. Biological Systems

Biological systems are based on strategies that can compete in field environments with toxic fungi. Different microorganisms have been reported as bio-control agents of *A. flavus* and aflatoxin in pre-harvest; for example, Dorner and Cole [51] showed that the treatment of soil with non-toxic strains of *A. flavus* and *A. parasiticus* significantly reduced aflatoxin contamination. In addition, Cleveland et al. [52] reported that soil treatment with atoxic *F. verticillioides* had the beneficial carry-over effect of excluding fumonisin-producing strains and preventing them from producing fumonisins. Luongo et al. [53] also described reduction of saprophytic colonization and sporulation of toxic *F. verticillioides* and *F. proliferatum* in maize residues by non-pathogenic *Fusarium* species. The ability of fungal antagonists to cope with toxic fungi is, however, related to macro- and micro-climatic conditions in the antagonist–pathogen interaction. The efficiency of mycotoxin bio-control agents depends on their ability to colonize the target substrate and to be active under different environments in the field or during storage without inducing effects that compromise the end use quality of the commodity [54]. Insect herbivory creates wounds on the corn kernels and acts as a vector for certain types of fungal spores [55]. In several field studies, transgenic Bt corn (containing a gene from the soil bacterium *Bacillus thuringiensis*, which encodes for production of a crystal protein that is toxic to common lepidopteran corn pests) has been shown to significantly lower the levels of common mycotoxins compared to non-Bt isolines [56,57,58,59]. These results indicate that it indirectly controls for one of the most important predisposing factors of mycotoxin accumulation (i.e., pest attack).

#### 2.1.3. Chemical Systems

Insect damage is well recognized as a collateral factor in mycotoxin development. Fungal colonization or insect infestation of crops must be controlled by appropriate use of registered insecticides, fungicides, and other proper practices within an integrated pest management control [60]. In particular, the application of fungicides and pesticides (e.g., benomyl, thiabendazole, prochloraz, dichlorvos, landrin, mathion, diazinon [61]) during the production process could assist in minimizing the fungal infection or insect damage of crops and consequently mycotoxin contamination. However, in both laboratory studies with pure cultures of pathogens and field trials with crop plants, the resulting evidence concerning the effectiveness of fungicides is contradictory and in certain cases somewhat unexpected. In a number of instances, fungicide concentrations tested in laboratory studies were in excess of maximum solubility levels in aqueous media and, therefore, the interpretation of results is not straightforward. Similarly, if chemical control is to succeed in the future, additional criteria may need to be introduced into evaluation protocols for candidate pesticides [61]. Furthermore, a more environmentally acceptable solution to the question of mycotoxin contamination of plant products needs to be exploited.

### 2.2. Post-Harvest Strategies

Although pre-harvest approaches should be preferred, in the perspective of preventing mycotoxin contamination, the development of toxic fungi is inevitable under certain environmental conditions. Therefore, appropriate storage practices and other post-harvest control systems are necessary to minimize the final mycotoxin content of foods and feeds. Several strategies are available for the fungal inactivation and mycotoxin degradation. These can be classified as (micro)biological, physical and chemical approaches.

#### 2.2.1. Storage Management

Appropriate storage methods may prevent mold growth, and optimal temperature and humidity of store houses can also reduce mold growth and prevent mycotoxin production (as reported before; [17]). A relatively low oxygen (O_2_) and high CO_2_ concentration contributes to the avoidance of fungal development and mycotoxin production [62]. Decreasing the O_2_ concentration (from 5 to 1%) can decrease *Aspergillus* growth and aflatoxin production [63]. Since gas composition is contemplated as one of the most important abiotic conditions that impacts fungal and pest growth, the use of a controlled atmosphere with a very high nitrogen (N_2_) concentration is a valid method to control grain quality post-harvest. Recently, Moncini et al. [64] documented that growth and sporulation of *F. graminearum*, *F. langsethiae*, *A. flavus* and *F. verticillioides*, and aflatoxins production by *Aspergillus* were significantly decreased when artificially contaminated corn and wheat grains were exposed to a highly purified N_2_ controlled atmosphere (98.5% ± 0.5). These results indicate that a N_2_-controlled atmosphere acts as an eco-friendly tool that could be transferred to a large-scale system for grain storage in order to avoid or reduce chemical treatments. Many of the above strategies are applicable to prevent toxic fungal development and mycotoxin formation. However, once mycotoxin contamination has occurred, feed and food associated with various mycotoxins must be managed through post-harvest detoxifying procedures.

#### 2.2.2. (Micro)biological Systems

Biological approaches for mycotoxin decontamination by using microorganisms (such as bacteria, fungi, and enzymes) have been used efficaciously for the management of mycotoxins in feed and food. Mycotoxin-degrading bacteria have been isolated from various sources like rumen and intestinal flora, soil, and even water. Improved feed utilization due to interactions between lactic acid bacteria (LAB) strains and mycotoxins (such as aflatoxin, ZEA, OTA and patulin) have been reported [65,66]. In particular, these bacteria prevent the growth of molds and consequently mycotoxins via specific hydrolytic enzyme production that decomposes carbohydrates and increases host’s enzyme activity. In addition, many mycotoxins can be adsorbed by LAB. The binding appears to be physical and is related to the bacterial surface [67]. Yeasts have also been proven to be useful for inhibiting some toxic fungal growth and to prevent mycotoxin biosynthesis. *Saccharomyces cerevisiae* and *Pichia carribica* showed high potentiality to degrade patulin as a less toxic compound during fermentation in juices, as well as to inhibit patulin production by *Penicillium expansum* during post-harvest [68,69]. Similarly, *Trichosporon mycotoxinivorans* can deactivate OTA into the nontoxic form after incubation for 2.5 h. Molnar et al. [70] developed this system into a commercial detoxifying OTA product for animal feed. For this reason, *S. cerevisiae* has been reported as the most efficient microorganism for AFB_1_ adsorption, since yeast cell walls allows the cells to adsorb a range of compounds from the environment [71]. In addition, esterified glucomannan polymer extracted from the *S. cerevisiae* cell wall was shown to bind T-2 [72]. Biocontrol is not restricted to bacteria and yeasts: molds such as *Aspergillus*, *Penicillium*, and *Rhizopus* are also able to detoxify mycotoxins; *Clonostachys rosea* can be used as a biocontrol agent in cereals, where it can produce zearalenone lactonohydrolase, thus avoiding other fungal growth on zearalenone-contaminated media [73]; and *Aureobasidium pullulans* can inhibit OTA accumulation and decrease aspergillosis occurrence and was therefore used as a biocontrol agent in wine grapes [74]. In addition, specific enzymes such as oxidase, peroxidase (EC 1.11.1.7), laccase (EC 1.10.3.2), oxidoreductase (EC 1), esterase (EC 3), carboxylesterase (EC 3.1.1.1), aminotransferase (EC 2.6.1), and lactonohydrolase (EC 3.1.1), having the capacity of degrading mycotoxins, have been purified from microbial systems [75]. A peroxidase from *A. flavus* and *A. parasiticus* and a horseradish peroxidase from *Raphanus sativus* showed AFB_1_ degradation activity. A purified extracellular enzyme, myxobacteria aflatoxin degradation enzyme, from the bacterium *Myxococcus fulvus* ANSM068 showed much degradation ability toward AFG_1_ and AFM_1_ (97 and 96%, respectively). *Brevibacterium* species are capable of degrading OTA, due to the release of highly active proteolytic carboxypeptidase enzymes (EC 3.4). A recent report revealed that OTA was significantly (75–85%) reduced by a carboxypeptidase and peptides present in liquid cultures of *Bacillus subtilis* CW14 [62].

#### 2.2.3. Physical Systems

Physical methods of mycotoxin decontamination include various procedures such as drying, cleaning, mechanical sorting and separation washing, thermal inactivation, and irradiation. Rapid drying of agricultural products reduces the moisture level, creating less favorable conditions for fungal growth and proliferation, and insect infestation [76]. Hamiton [77] reported that drying harvested maize to 15.5% moisture content (or lower) within 24–48 h, decreased the risk of fungal growth and aflatoxin production. Awad et al. [78] demonstrated that a moisture level of 6.6% in groundnuts effectively suppressed the cross infection (due to *A. parasiticus*) of healthy kernels. During storage, transportation and marketing, low moisture levels should be maintained by avoiding leaking roofs and condensation arising from inadequate ventilation. A study conducted by Fandohan et al. [79] demonstrated that treatments like sorting, winnowing, washing (and drying), crushing combined with de-hulling of maize grains were effective in achieving significant mycotoxins removal. This approach is based on the separation of contaminated grain from the bulk and depends on the heavy contamination of only a small fraction of the seeds, so that removing those leaves a much lower overall contamination. In fact, it was demonstrated that the major portion (approximately 80%) of the toxin is associated with the small and shriveled seed. Similarly, Varga, and Tóth [34] reported that a simple washing (and then drying) procedure (using water or a solution of sodium carbonate) reduces the concentration of some mycotoxins in grains or corn cultures. It is worth pointing out that this might be a useful procedure only prior to wet milling, because of the eventual high price of the subsequent drying procedures [80]. Thermal inactivation is not a suitable method for decontamination, because most of the mycotoxins are quite heat stable. However, microwave radiation can be used in the detoxification of aflatoxin in groundnut meal and peanuts [81,82], or trichothecenes in corn [83]. Ultraviolet (UV) irradiation is a potential non-thermal technology for the decontamination of mycotoxins [84]. Irradiation technologies are usually applied in feed and food industry because of their high efficiency to eliminate the microorganisms and other potential pathogens infecting the grains. Both heating and hydrolysis are the main mechanisms involved with inhibition of fungal growth and aflatoxins, T-2 toxin or DON reduction by irradiation (when applied to a thin layer of grains; [85]). The growing literature reveals that cold or non-thermal plasma is another effective tool for the degradation of mycotoxins [86].

#### 2.2.4. Chemical Systems

Numerous chemical agents are suitable for mycotoxin control involving bases (such as ammonia and hydrated oxide), oxidizing agents (e.g., hydrogen peroxide and O_3_), organic acids (formic and propionic acid) and other agents. Ammonization of grains not only reduces aflatoxins, fumonisins, and OTA to undetectable levels, but also inhibits toxic fungal growth. However, this method is not permitted in the European Community for human foods [87]. Recently, a mixture of glycerol and calcium hydroxide was shown to have a powerful detoxification effect on mycotoxins [88]. Chemical decontamination methods have already been accepted for use in industry, but more novel detoxification methods must be developed and investigated for use in agricultural products while considering public concerns about animal feed and human food. Recently, some authors have proposed the removal of mycotoxins using nanoparticles (e.g., magnetic carbon nanocomposites, silver and chitosan-coated Fe_3_O_4_ nanoparticles), essential oils and their main bioactive compounds (e.g., clove oil and its major ingredient, eugenol). These novel techniques are apparently promising, effective, and low-cost strategies that can offer eco-friendly solutions for the control of toxic fungi and mycotoxins in agricultural and food industries [67], but further validation is needed, and doubts remain about the quantity of essential oils needed for this type of treatment. The strong desire to reduce the use of chemicals applied in the food and feed chains and the lack of effective and straightforward solutions to control mycotoxin contamination in the field, at harvest, and of processed products, leads to the demand for methods for their partial or total elimination.

Ozonation is a simple technology, which does not leave harmful residues after application. The use of O_3_ in the degradation of several mycotoxins has been reported in many papers [89,90,91,92,93]. Ozone is used as an oxidizing agent to disinfect cereals, vegetables, and fruits, or to detoxify mycotoxins [89]. However, limitations and gaps in knowledge about this method in mycotoxin detoxification have been highlighted. All these aspects related to the use of O_3_ to control mycotoxins in feed and food are scrutinized in the sections below.

## 3. Mycotoxin Detoxification in Food and Feed by Ozone

### 3.1. Ozone Applications in Food/Feed Processing

Ozone, an odd molecule made of three oxygen atoms, is a gas that occurs naturally in very small amounts in the Earth’s atmosphere, both in the Earth’s upper atmosphere and at ground level [94]. High in the stratosphere, about 25 km above the Earth, O_3_ is formed naturally in the so-called “O_3_ layer” from atmospheric oxygen by exposure to UV light and serves as a protective layer that absorbs harmful UV radiation (“friend O_3_”). But human beings make extra O_3_ every day (“foe O_3_”). This ground-level O_3_ is formed when exhaust gas emissions from vehicles (mainly represented by nitrogen oxides and volatile organic compounds) interact with sunlight, especially in the summer; the entire complex of reaction is known as “photochemical smog”. When O_3_ is above threshold levels (e.g., air quality standards for the protection of health, as given in the EU Ambient Air Quality Directives set the O_3_ target value at 120 µg m^−3^ as a maximum daily 8-hour mean, not to be exceeded on more than 25 days a year^−1^, averaged for three years [94]), it can irritate lungs and cause severe short- and long-term health problems. 

Being made up of three fairly stable oxygen atoms, O_3_ is a powerful oxidizing unit and therefore a potent disinfecting agent, and its use may have many advantages in the food industry, such as in food surface hygiene, sanitation of food plant equipment, reuse of waste water, disinfection of packaging materials, and control of noxious organisms in stored products. Microbicidal action of O_3_ is gaining attention due to the fact of zero residues on the product and no need of aeration to remove the gas [95].

Ozone can be generated starting from dry oxygen through machineries, and the electric corona discharge or UV radiation are the mostly used methods for O_3_ generation at a commercial level [95]. However, the electric corona discharge is usually more useful and inexpensive to obtain high concentrations of O_3_. Ozonisers adopting this method expose O_2_ molecules to a high voltage of electrical discharge, so initiating the formation of free radical O_2_ and thereby generating O_3_ [96]. Breakage of the O–O bond can also be accomplished by photochemical, radiochemical, thermal, chemonuclear, and electrolytic methods [97]. 

**Figure 2:**
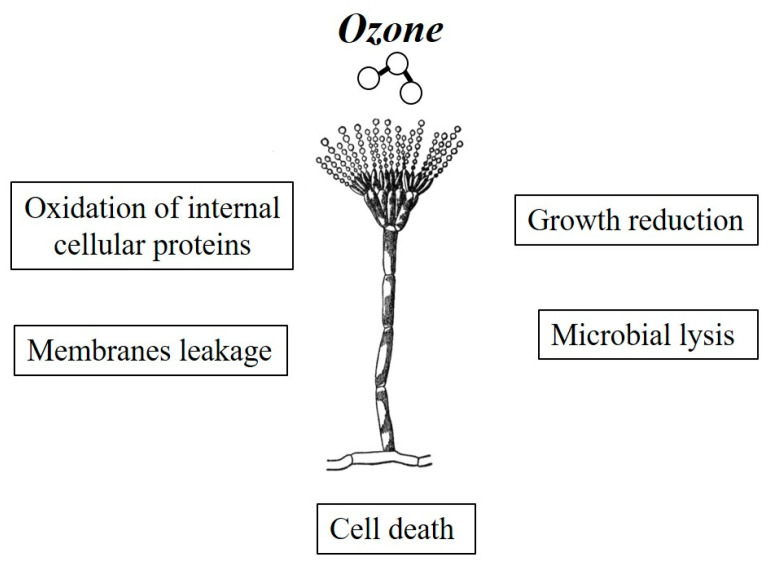
A summary of ozonation effects on fungal cells resulting in their inactivation.

Ozone, discovered in the atmosphere by Schonbein in 1839, was approved by the Food and Drug Administration (FDA) to be Generally Recognized as Safe (GRAS) for bottled water disinfection early in 1982 [98], and its use was extended to food treatment, storage, and processing in 1999 [99]. In 2001, O_3_ was formally approved as an antimicrobial agent for the sanitization of foods in order to tackle the environmental and occupational contamination related to the use of chlorine [100]. Indeed, this use does not leave any residues in food since O_3_ merely dissociates into O_2_ and related radicals. For this reason, the application of O_3_ technology in food chains has been considered safe and effective by the WHO, and is now recognized as a “green technology” [101]. With a redox potential of 2.07 V, O_3_ must be considered one of the most powerful oxidant disinfectants, being 1.5- and 1.3-fold stronger than chlorine and hydrogen peroxide against bacteria, virus, algae, and fungi [102]. Ozonation is thus used as a physico-chemical mean for microbial inactivation in food/feed processing, applied on fresh products such as fruits and vegetables, liquid foods such as juices, dairy products such as milk and cheese, food derivatives such as flours and poultry, as well as their intermediates [103]. The mechanisms involved in microbial inactivation are complex. Several studies have documented that O_3_ is able to disable noxious organisms such as pathogenic microbes and storage pests, by oxidizing their vital cellular components. In particular, O_3_ acts against unsaturated lipids in the microbial cell membranes causing a leakage of their contents, and, eventually, microbial lysis [104]. In addition to this damage, O_3_ causes widespread oxidation of internal cellular proteins reducing their growth and causing rapid cell death (Figure 2; [95]). As reported above, a major advantage of ozonation is that all O_3_ decomposes to produce O_2_, leaving no residue in food/feed. Therefore, O_3_-treated products are safe for consumption and their microbiological shelf life can be greatly enhanced [105]. However, O_3_ efficacy in food/feed processing depends on several factors such as the method, concentration and timing of the O_3_ application, the microorganisms/contaminants to inactivate, and the type of food/feed processed. Thus, it is worth keeping in mind that food/feed ozonation is not always a beneficial process, since O_3_ can also cause alterations in food/feed such as changes in sensory characteristics, color loss, lipid oxidation, and the degradation of phenolic compounds and vitamins [106]. For this reason, it is important to study not only the effect of ozonation on mycotoxin degradation, but also the effect of this method on overall product quality [90,91,107,108,109].

Ozone can be directly applied to food/feed in its gaseous form or dissolved in aqueous solutions and applied as ozonized water. In the gaseous form, the half-life of O_3_ is a few hours in the presence of food/feed. When bubbled into water, O_3_ dissolves, partially forming hydroxyl radicals that can oxidize the microorganisms/contaminants more efficiently than molecular O_3_ itself [75]. However, its solubility in water is dependent on several factors such as partial pressure, temperature and the pH of water, as well as its purity, since the presence of minerals and organic matter can “consume” O_3_ [110]. Ozonized water is especially suitable for raw materials such as corn, wheat grains, and flour that require an aqueous disinfection step, as well as for fruits and vegetables that must be washed [101], but the O_3_ gaseous form is considered the more useful application in decontaminating mycotoxins. Both methods have been successfully used to reduce postharvest diseases, viability of toxic fungi and mycotoxin accumulation in food/feed products. The last review on the application of O_3_ to prevent and degrade mycotoxins dates back to 2010. Here, an updated knowledge of the topic acquired in the last ten years is reported.

### 3.2. Ozone: A Powerful Tool to Detoxify Mycotoxins (?)

**Figure 3:**
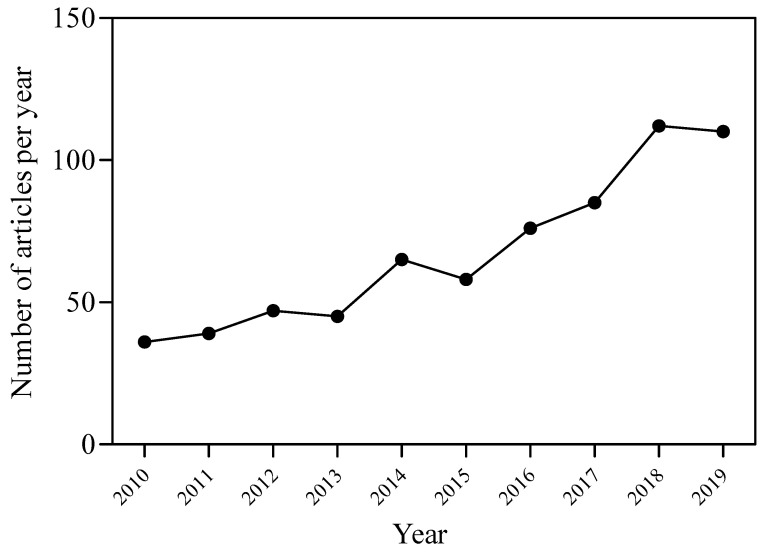
Distribution of relevant studies (total = 673) published between 2010 and 2019, selected using the online versions of ScienceDirect and Scopus, and searching for the terms “ozone” or “oxidant agent”, and “mycotoxins”, “aflatoxins” or “trichothecenes”, and “food” or “feed”.

The frequency distribution of studies published between 2010 and 2019, selected using the online versions of ScienceDirect and Scopus databases (https://www.sciencedirect.com/ and https://www.scopus.com/), and searching for the terms “ozone” or “oxidant agent”, and “mycotoxins”, “aflatoxins” or “trichothecenes”, and “food” or “feed”, is shown in Figure 3. The number of papers from 2020 is 57 and is not comparable with previous years since only five months need to be taken into account (January–May). Since 2010, some reviews on the application of O_3_ as a strategy to prevent/degrade mycotoxins have been already published [4,102]. Some of these reviews focused on the use of O_3_ as a decontamination tool during the processing and storage of many food products, including cereal grains [111,112,113,114], citrus fruits and derivatives [115] and dried fruits [116], and feedstuffs [117,118,119,120]. The attention of the scientific community on O_3_ technology seems to be increasing, as demonstrated by the big rise in studies published in the last few years (Figure 3). These data highlight the full potential and interest reported for the mycotoxins detoxification of contaminated agricultural products by O_3_ treatment (Table 1).

According to these studies, there is no doubt that ozonation is an easy technology and is effective at decontaminating mycotoxins. The application of gaseous O_3_ was reported as more useful and most of the reviewed experiments (79%) documented the higher effectiveness and the practical advantages of a gaseous application of O_3_ than aqueous solutions. This method is a more useful type of application and it has been successfully used in reducing mycotoxin accumulation in many kinds of food/feed products (e.g., cereals [121,122,123], dried fruits [124], and poultry [125]). The residual 21% were experimental studies focused on demonstrating the suitability of ozonated water for the mycotoxin inactivation in raw materials that require an aqueous disinfection step and that must be washed [90,126,127,128,129,130].

As reported above, ozonation is a chemical mean for food/feed processing, that has been used on many kinds of products or intermediates. Most of the reviewed experiments (81%) were carried out on cereals, in particular wheat and corn, and their derivatives such as flour [90,91,121,128,131,132], malting [122], and co-products like pasta and semolina [92]. Another large percentage of studies (19%) was conducted on dried fruits/seeds (in particular peanuts and pistachios).

**Figure 4:**
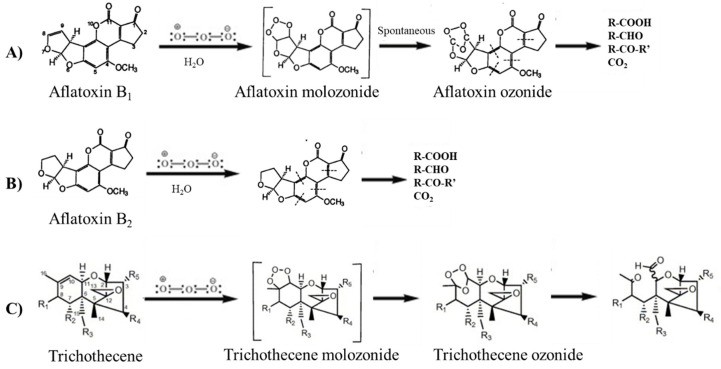
Proposed mechanism for the addition of ozone to aflatoxin B_1_ (**A**) and B_2_ (**B**) and trichothecene (**C**).

Of the approximately 400 compounds identified as mycotoxins, 30 are considered a threat to human and animal health [4]. Most of the reviewed experiments (50%) documented that gaseous O_3_ was able to degrade aflatoxins in many food/feed portions and in several operation conditions (e.g., higher O_3_ concentration and/or longer treatment [133], higher temperatures [127,134], initial level, and type of contamination). In particular, O_3_ was reported to be successful in the degradation of AFB_1_ and AFG_1_, since there is a C8–C9 double bond forming the vinyl ether at the terminal furan ring in their structures, which is not present in AFB_2_ and AFG_2_ [135]. The degradation of AFB_2_ and aflatoxin G2 (AFG_2_) requires longer O_3_ exposure until the lactone ring is opened by the O_3_ treatment (Figure 4A,B; [93]). Specifically, AFB_1_ is the most harmful of the four naturally occurring aflatoxins because of its remarkable hepatotoxicity and carcinogenicity (it has been classified as Group I of human carcinogens [136]). Consequently, this food/feed contaminant has been the focus of all reviewed experiments. Torlak et al. [125] reported that considerable reductions can be achieved in the levels of AFB1 in poultry feed (composed of corn, soybean and sunflower meal, barley, limestone, dicalcium phosphate, salt, vitamin/mineral pre-mix, and methionine) by gaseous O_3_ treatment without causing significant increases in lipid oxidation.

Another large percentage of studies (30%) was conducted on trichothecenes type A such as HT-2 and T-2 toxins, and type B such as DON [101,135] in many commodities and in several operation conditions (e.g., higher O_3_ concentration and/or longer treatment times [109,110,137,138], moisture [90], initial level, and type of contamination). In particular, it has been documented that the degradation of 10 trichotecenes began with an attachment of O_3_ to 9,10 double bond with the net addition of two atoms of O_2_, with the remainder of the molecule left unaltered (ozonolysis, or “Criegee mechanism”; Figure 4C; [139]). A small percentage of these studies (3%) was devoted to investigating the effectiveness of O_3_ treatment in detoxifying/degrading ochratoxins [140]. The remaining 17% of the studies was conducted on ZEN (produced by the fungi of the *Fusarium* genus [141]) and citrinin (produced by a wide variety of *Penicillium* species [134]). It is worth noting that few studies have evaluated the simultaneous O_3_ decontamination of different mycotoxins in relation to their potentially additive or synergistic combination [109,123,124,132,134,142,143,144]. This problem has presented an ardent challenge to the development of uniform methodologies for detoxification of foods/feeds. Although a decontamination protocol may effectively reduce the detectable levels of one toxin, another toxin may cause the production of biologically active compounds that maintain a toxic or a mutagenic potential [145,146].

## 4. Conclusions and Future Perspectives in the Use of Ozone for Detoxification

The present review paper on mycotoxin contamination of feed and food highlights (i) the lack of effective and straightforward solutions to control mycotoxin contamination in the field at pre-harvest, as well as later at post-harvest, and (ii) the increasing demand for novel methods to control mycotoxin infections, intoxications, and diseases, without leaving toxic chemical residues in the food and feed chain.

Ozone application has given promising results for important problems in the food industry, such as mycotoxin and pesticide residues. In particular, O_3_ is able to inhibit fungal growth, sporulation and germination, by offering negligible loss of nutrients or sensory qualities in food/feed. However, its antimicrobial activity is very dependent on vegetable/fungus species, growth stage, concentration, and exposure time. In addition, degradation products, formed after O_3_ treatment of these residues, have not exactly been determined, and this seems to be the most crucial obstacle on this subject. In vivo and in vitro toxicological tests should be conducted to screen the effects of degradation products on human and animal health. Through emerging new techniques (reactive oxygen/nitrogen species), as well as improvements and innovations in O_3_ generation and application systems, certainly the subject will be evaluated more effectively in the future, facilitating enhanced control of both quality and safety parameters of ozonized foods/feeds. For effective and safe use in processing, optimum O_3_ concentration, contact time, and other treatment conditions should be defined for foods and feeds. A pilot test must be conducted for each case before starting a commercial application, since every O_3_ application is unique. Industrial facility of O_3_ technology remains to be developed for large scale treatment of food/feed products, requiring input from different disciplines.

## Figures and Tables

**Table 1:** Ozone (O_3_) effects on mycotoxins degradation in different food/feed commodities.

Year	O_3_ Concentration/Application	Food/Matrix	Target	O_3_ Main Effect	References
2011	26 g m^−3^ of gaseous O_3_ for 120 min after 2 and 6 h of steeping	Malting barley	Deoxynivalenol (DON)	No reduction (probably due to the low initial concentrations of DON)	[122]
2012	13 and 21 g m^−3^ of gaseous O_3_ for 24, 48, 72, and 96 h	Peanuts kernels	Aflatoxin B1 (AFB_1_)	Reduction of 25% after 96 h of exposure at 21 g m^−3^ of O_3_	[142]
2012	20 and 40 g m^−3^ of gaseous O_3_ for 5, 10, 15, and 20 min	Wheat grains	AFB_1_	Total reduction of AFB_1_ after 10, 15 and 20 min of exposure	[145]
2013	50 g m^−3^ of gaseous O_3_ for 60 h	Peanuts	AFB_1_	Reduction of 89%	[124]
2013	4 and 8 g m^−3^ of aqueous O_3_ for 2, 4, 6, 8, 10, and 12 h	Pistachio kernels	AFB_1_	Reduction by 48, 13, 46, 44 and 44% after 2, 4, 6, 8, 10 and 12 h	[130]
2014	15, 30, 45, and 75 g m^−3^ of gaseous O_3_ for 60 min	Corn flour	AFB_1_, Aflatoxin B2 (AFB_2_), Aflatoxin G1 (AFG_1_) and Aflatoxin G2 (AFG_2_)	Reduction of AFB_1_, AFB_2_ and AFG_1_ by 79, 71 and 72% at 75 g m^−3^	[132]
2014	90 g m^−3^ of gaseous O_3_ for 20 and 40 min	Corn	AFB_1_	Reduction by 78 and 88% after 20 and 40 min of exposure, respectively	[143]
2014	79 and 118 g m^−3^ of gaseous O_3_ for 30, 60, 120, and 180 min at room temperature	Wheat grains	DON	Reduction to the limit of detection in wheat grains after 120 min of exposure at 118 g m^−3^	[108]
2014	6.0 g m^−3^ of gaseous O_3_ for 30 min at room temperature	Peanuts	AFB_1_	Reduction of 66%	[134]
2015	79 and 118 g m^−3^ of gaseous O_3_ for 30, 60, 120, and 180 min at room temperature	Wheat grains	AFB_1_, AFB_2_, AFG_1_, AFG_2_ and citrinin	Concomitant reduction of AFB_1_, AFB_2_, AFG_1_, and AFG_2_ by 95, 85, 80, and 81% in wheat grains after 180 min of exposure at 118 g m^−3^, respectively. Under the same O_3_ concentration, reduction of citrinin by 29, 45, 46, and 75% after 30, 60, 120, and 180 min	[133]
2015	1, 2 and 2.5 g m^−3^ of aqueous O_3_ for 60, 120, and 180 min at 20, 25, and 40 °C	Wheat	AFB_1_, AFB_2_, AFG_1_ and AFG_2_	Reduction of AFB_1_ by 27, 34, and 40% in wheat samples after 180 min of exposure at 2.5 g m^−3^ of O_3_ (at 20, 25, and 40 °C, respectively). AFG_1_ and AFG_2_ were completely inhibited when samples were treated with 2 and 1 g m^−3^ of aqueous O_3_, respectively	[126]
2015	10 m g^−3^ of gaseous O_3_ for 30 s	Wheat	DON	Reduction by 94%	[89]
2016	8.5, 13.5, 20, 25, and 40 g m^−3^ of gaseous O_3_ for 20 min	Aflatoxins dissolved in water	AFB_1_, AFB_2_, AFG_1_ and AFG_2_	Rapid elimination of AFB_1_ and AFG_1_	[135]
2016	10 g m^−3^ of aqueous O_3_ for 15 min at room temperature	De-hulled dried pistachios	AFB_1_	No reduction	[129]
2016	100 g m^−3^ of gaseous O_3_ for 1 h at 20% moisture	Wheat flour	DON	Reduction by 78%	[127]
2016	75 g m^−3^ of gaseous O_3_ for 30, 60, and 90 min	Wheat flour	DON	Reduction by 26, 39, and 54% after 30, 60, and 90 min	[131]
2016	60 g m^−3^ of gaseous O_3_ for 300 min	Wheat grains	Aflatoxins and DON	Reduction of aflatoxins and DON by 64 and 48%, respectively	[101]
2016	20 g m^−3^ of gaseous O_3_ for 40 and 130 min	Wheat grains	DON, HT-2 toxin (HT-2), T-2 toxin (T-2) and zearalenone (ZEA)	Reduction of HT-2, T-2 and ZEN by 65, 62, and 59% after 40 min. Reduction of DON by 25% after 130 min	[138]
2016	2.8 and 5.3 g m^−3^ of gaseous O_3_ for 240 min at room temperature	Poultry feed composed of corn, barley, soybean and sunflower meal	AFB_1_	Reduction by 74 and 86% at 2.8 and 5.3 g m^−3^ of O_3_	[125]
2016	100 g m^−3^ of gaseous O_3_ for 180 min	Corn	ZEA and OTA	Reduction of ZEA and OTA by 91 and 71%, respectively	[123]
2016	80 g m^−3^ of aqueous O_3_ for 10 min	Contaminated Wheat, corn and bran	DON	Reduction by 75, 71, and 76% in contaminated wheat, corn, and bran, respectively	[127]
2017	65 g m^−3^ of aqueous O_3_ for 60, 120, and 180 min at 10 and 25% of moisture	Wheat flour	DON	Reduction by 70, 70, and 78% in wheat flour after 60, 120 and 180 min of exposure at 25% of moisture	[90]
2018	62 g m^−3^ of gaseous O_3_ for 15, 30, 60, 120, 180, and 240 min	Wheat bran from contaminated grains	DON and ZEA	Reduction of DON by 29, 45, and 32% in wheat bran after 15, 30, and 240 min of exposure. Reduction of ZEA by 57 and 61% after 15 and 240 min treatment	[109]
2018	40 g m^−3^ of gaseous O_3_ for 6 h	Wheat grains, semolina and pasta	DON and DON-3-Glc	Reduction of DON and DON-3-Glc by 29 and 44%	[92]
2019	52 g m^−3^ of gaseous O_3_ for 5, 10, 20, 30, and 60 min	Maize flour	ZEA	Reduction by 38, 56, and 62% in maize flour after 5, 10, and 60 min of exposure	[91]
2019	20–60 g m^−3^ of gaseous O_3_ for 120–480 min	Corn grits	AFB_1_, AFB_2_, AFG_1_ and AFG_2_	Reduction of AFB_1_, AFB_2_, AFG_1_, and AFG_2_ by 55, 57, 36, and 30% after 480 min of exposure at 60 g m^−3^	[93]
2019	10 g m^−3^ of gaseous O_3_ for 30 s	Scabbed wheat	DON	Reduction by 94%	[138]
2019	50 g m^−3^ of aqueous O_3_ for 90 min	Corn flour	ZEN	Reduction by 95%	[128]
